# Supplementary material for: Activation in human auditory cortex in relation to the loudness and unpleasantness of low-frequency and infrasound stimuli
Source: PLoS One. 2020 Feb 21;15(2):e0229088. doi: 10.1371/journal.pone.0229088 (PMC7034801; doi:10.1371/journal.pone.0229088)
Supplement: S1 Fig — (PDF) [file pone.0229088.s001.pdf]

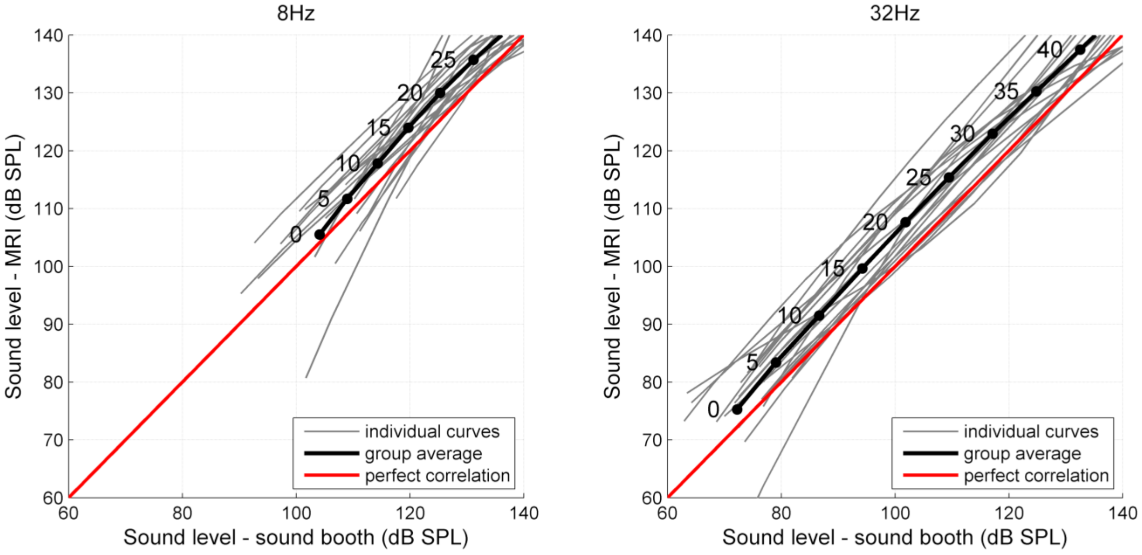

S1 Figure. Comparison of loudness functions in the MRI environment versus the sound booth.

Results are shown for the 8 Hz tone (left) and the 32 Hz tone (right). Sound pressure levels corresponding to all categorical loudness units extracted from functions fitted to loudness judgments obtained in the fMRI experiment are plotted against the respective levels from judgments obtained in the sound booth. The light grey curves represent comparisons at the individual (participant) level, the black line represents the average across all individual data. For the averaged data, the levels corresponding to specific categorical loudness units (0 to 25 and 0 to 40 for 8 Hz and 32, respectively) are highlighted. For comparison, the red line depicts a theoretical perfect correlation between both origins of loudness functions.

Group averaged loudness functions fitted to the judgments obtained in the MRI experiment are almost linearly related to those in the sound booth, except for a small offset in level. Specifically, slightly higher levels were required in the MRI scanner to elicit the same loudness perception. Individual functions vary in steepness and level offset. An extreme deviation can be seen for one participant, whose estimated detection thresholds (~ 0 cu) are almost 20 dB lower in the noisy MRI environment than in the silent booth, which appears at least questionable.
