# Supplementary material for: Activation in human auditory cortex in relation to the loudness and unpleasantness of low-frequency and infrasound stimuli
Source: PLoS One. 2020 Feb 21;15(2):e0229088. doi: 10.1371/journal.pone.0229088 (PMC7034801; doi:10.1371/journal.pone.0229088)
Supplement: Attachment — Submitted filename: PloS_One_Revision1.docx [file pone.0229088.s002.docx]

**We’d like to thank the reviewers for the constructive and useful feedback on the previous version of the manuscript. In our view, the changes applied on the basis of the reviewers’ comments have considerably improved the quality of the manuscript. Below, the comments made by the reviewers are duplicated in plain font, together with our responses in bold font. Added or changed text taken from the revised manuscript is given in italics. Note that some minor changes to the manuscript, such as text rewordings and fixed typos, might not be explicitly listed here. All line numbers refer to the initial version of the manuscript.Yours sincerely,**

**Oliver Behler and Stefan Uppenkamp**

5. Review Comments to the Author

Please use the space provided to explain your answers to the questions above. You may also include additional comments for the author, including concerns about dual publication, research ethics, or publication ethics. (Please upload your review as an attachment if it exceeds 20,000 characters)

Reviewer #1: In the current study, Behler & Uppenkamp examined behavioral and neural responses to acoustic stimuli with varying veridical loudness, perceived loudness, and perceived pleasantness. To this end they presented healthy participants with low frequency sounds (LFS; < 200Hz) and infrasonic sounds (IS; < 20Hz) monaurally to the right ear. The stated aims of the study are:

(1) Are perceived loudness and unpleasantness distinctly represented in the human brain?

(2) Can interindividual differences with respect to loudness and unpleasantness for LFS and IS be identified in terms of objective, physiological correlates?

Throughout the paper, the stated aims get mixed (and side-tracked) with analysis focusing on alternative questions.

The authors go to a lot of effort to show that the perceptual curves outside/inside the scanner are similar (which is interesting by itself), and dedicate a lot of effort in the comparison of 8Hz and 32Hz stimuli. These are different questions from the stated framework of the study that left me confused with respect to what is the scientific question that the authors are trying to answer.

**Response 1.1: Possible differences between LFS (at the lower limit of pitch perception) and IS (perceived as “seperate puffs”) in terms of their perception and neural representation were in fact also a central research question in this study. However, we acknowledge that this was not sufficiently stated yet. We have now added a third numerated item to the respective section in the introduction to clarify this (lines 60 ff.):**

***(1) Do perception and neural responses differ between LFS (at the lower limit of pitch perception) and IS tones? (2) Are perceived loudness and unpleasantness distinctly represented in the human brain? (3) Can interindividual differences with respect to loudness and unpleasantness for LFS and IS be identified in terms of objective, physiological correlates?***

**With regard to the comparison of loudness functions across different environments, on the other hand, we agree with the reviewer’s comment below that the results are important, yet subsidiary with respect to the stated aims. For the purpose of streamlining the manuscript, we have moved the corresponding figure (formerly Figure 3) to the supporting information (now S1 Figure). The first sentence of the paragraph that briefly describes the findings of this comparison (lines 304-305) has now been moved to the end of the first paragraph of the results section. The remaining text (lines 305-309) which describes the results in more detail has now been removed from the manuscript and part of that has been added to the supplementary figure caption.**

Another example - what is the logic of presenting sounds monaurally? Is this part of the author’s question (bilateral vs. contralateral activations)? When the question is unclear, the logic of the experimental design is also unclear and the focus of the paper gets lost.

**Response 1.2: Stimuli were delivered directly to the ear canal instead of using free-field (whole body) stimulation since we were specifically interested in how LFS and IS are processed by the auditory system only. We also wanted to avoid possible confounds due to other bodily reactions at high levels. This motivation is now explicitly stated in the discussion of the revised manuscript (line 654). Apart from that, free-field stimulation would not have been possible due to fMRI restrictions.**

**The presentation of monaural instead of binaural stimuli was mainly due to technical restrictions. There was only one sound source available, which was located outside the MRI room and was coupled to one ear via a long tube. At the time of the measurements, there was no way to control the delivery of the stimuli to both ears with the same level and with no interaural phase difference.**

**The lateralization of activation was in fact not a central topic in the design of the study. We acknowledge that this topic probably took up too much space in the previous version of the manuscript, which distracted from the central questions. In the greater scheme of “re-framing“ the manuscript, as suggested by the reviewer in another comment, the information in the results section about the lateralization of activation has therefore been cut down to just a little more than one sentence. By contrast, we advocate keeping the single short paragraph devoted to lateralization in the discussion (lines 546 ff.), which has now been slightly reworded. In our view, this information may be valuable to other researchers, although we cannot yet provide an adequate explanation for it. Two additional mentions of a contralateral dominance of significant clusters (lines 584-585 and 590) have been removed.**

The presented data demonstrates:

1) Increase in absolute sound level (SPL) results in increased perceived loudness (figure 2). This is to be expected.

**Response 1.3: While this was indeed to be expected, the plots also show slight differences between 8 Hz and 32 Hz in terms of the growth of loudness with level, and the degree of interindividual differences with respect to loudness functions. Since these form the basis of potential differences between regression models in terms of explaining fMRI activation, it is in our view essential to present these data.**

2) There is correspondence between perceived loudness assessed outside/inside the fMRI scanner (figure 3). This is an important and novel observation to the best of my knowledge, although subsidiary with respect to the stated aims.

**See Response 1.1 and Response 1.6.**

3) Figure 4 suggests that IS are reported as more unpleasant than LFS although at the group level there is no difference.

**Response 1.4: In our view, Figure 4 does not explicitly suggest that IS is reported as more unpleasant than LFS in general. However, there is indeed no difference between IS and LFS at the group level if plotted against loudness. The group data only implicitly suggest that unpleasantness ratings grow more rapidly as a function of level for IS relative to LFS. This, in turn, can be inferred when considering that loudness grows more steeply with level for IS (which is shown in Figure 3). These issues are discussed already in the manuscript (lines 475-480).**

**Moreover, the plot shows the interindividual differences in terms of perception, with some participants reporting IS as more unpleasant than LFS at similar loudness values, yet others reporting the opposite. Lastly, it documents the number of (sometimes very few) available data points per subject, which may be important for the evaluation of the findings.**

4) Figures 5 & 6 & 7 demonstrate activation in auditory cortex to the 8 and 32Hz stimuli that increases with sound loudness.

**Response 1.5: While Figure 5 only showed significant responses to the average stimulus intensity for both frequencies, the other two figures, in fact, demonstrated an increase of activation with increasing level. The information provided by both figures was still slightly different, with one showing regions characterized by a positive linear relationship between BOLD signal and sound level (Figure 6), whereas the other only showed regions that were activated at two specific levels (Figure 7). Figures 5 and 7 have now been removed, to improve readability and minimize distraction from the main story of the manuscript (see also Response 1.6. below)**

5) Figures 8 & 9 pertain directly to the stated aims and suggest that perceived loudness and degree of pleasantness are more closely related to the degree of fMRI activation in auditory cortex as compared to absolute sound level.

I have no technical reservations but in my opinion a re-framing of the manuscript would help strengthen the manuscript and allow the authors to address their stated aims in a more focused manner.

**Response 1.6: We acknowledge that the number of different statistical analyses and results figures in the previous version of the manuscript was rather high and might have distracted the reader from the main focus of the study.**

**After critical re-evaluation of all reported analyses and data, we have decided to apply the following major changes to re-frame the manuscript**

1. **As stated in Response 1.1, we agree that the correspondence between loudness functions in different environments is an important finding, but we think that it may be safe to move the corresponding figure (Figure 3) to the supplemental material and to describe the respective results more sparsely in the manuscript.**
2. **The standard GLM analysis of the data in the first functional run and all corresponding results and references have been removed. The results of this analysis mainly served to show a) that 8 Hz and 32 Hz elicited similar responses in general and similar responses to sounds in the typical audiofrequency range. This can also be inferred from the data of the second functional run and is now reported accordingly; b) that activation in AC increases as a linear function of sound level. This can however also be inferred from the results of the cross-validation analyses (although this was not significant for the 8 Hz data alone, suggesting that this type of analysis might be less sensitive. Yet there is still a significant relationship between AC activation and loudness for 8 Hz). Plus, the extent of activation was obviously larger for the high intensity tones relative to the low intensity tones in the second functional run. By removing this analysis from the manuscript, we lose some details of putatively minor importance, such as an increase of activation in the anterior insula in response to the tones. This finding was however already discussed with great caution, since it was only seen for this particular type of analysis of data in the first functional run. The removal of this analysis from the manuscript required considerable changes to the text in the methods, results, and discussion sections of the manuscript.**
3. **As stated in Response 1.2, some of the results with respect to the lateralization of activation have now been removed from the manuscript.**

General comments:

1) A schematic figure showing when stimuli were delivered with respect to fMRI sampling and subject motor responses would be helpful.

**Response 1.7: The fMRI paradigm, especially with respect to the loudness scaling experiment, was, in fact, quite sparsely explained. The revised manuscript now includes the requested schematic figure (new Figure 2), as well as some important details about the loudness scaling procedure in the first functional run (lines 185 ff.):**

***“The task was modified so that tones were always presented four seconds after the onset of an image acquisition, with a jitter ranging from 500 to +500 milliseconds (see Figure XY, panel A). When participants did not complete the judgment of the last played tone before the onset of a scan, a silent trial followed and the presentation of the next tone was postponed to the subsequent interscan interval.”***

2) Titles for the figures could facilitate readability.

**Response 1.8: Titles for the figures were given in the figure captions (bold text), which are currently placed below the respective paragraph of their first mention in the manuscript, as required by the author guidelines.**

3) For the stated aims it seems that MEG (or some other method with a more acoustically quiet recording environment) would have been more appropriate.

**Response 1.9: There are certainly some advantages to the use of MEG, such as less acoustic background noise and higher temporal resolution, which also allows for other ways to approach the analysis of neural responses to LFS and IS sounds. Unfortunately, the MEG system at the University of Oldenburg was not available for this project at the time of this study., However, it is certainly something to be employed in future. Still, our data suggest that the background noise produced by the MRI scanner was not that much a problem, as we have seen that loudness perception was similar in the MRI environment and the sound booth.**

Reviewer #2: In this fMRI study, Behler and Uppenkamp investigated the neural basis of the audition of low frequency sounds (LFS, 32 Hz) and infrasounds (IS, 8 Hz) in relation to subjective estimates of sensory loudness and unpleasantness.

The authors found a high between-participants variability in detection thresholds, loudness functions and unpleasantness functions. On the neural level, the authors found significant tone-evoked activation patterns in the bilateral primary and secondary auditory cortices, which did not differ between 32 Hz and 8 Hz sounds and which increased as a function of sound physical level. Moreover, they did not observe a significant correlation between the interindividual differences in loudness or unpleasantness ratings and the variation of the fMRI activation. Within-participants differences in the bilateral activation patterns of the auditory cortices were predicted by sound levels, unpleasantness ratings and, especially, loudness ratings.

The study is well-designed, technically well-implemented and the results are interesting. However, I have some concerns which the authors should take into account:

[1] The first point is referred to the fMRI experiment. During the second functional run, participants were presented with 8 Hz and 32 Hz tones, whose low/high intensities were individually adjusted according to the loudness ratings provided by each participant during the first functional run. However, authors only collected unpleasantness judgements during the behavioural experiment, which was performed outside the MRI scanner (as stated in the Methods section 2.6.2). Even if the loudness functions in both conditions were linearly related, how can authors assume that the individual unpleasantness estimates in the noisy MRI environment (not collected) would have corresponded to those collected outside the MRI scanner (i.e. inside the silent booth)?

Moreover, in the Discussion the authors stated that “Normal hearing listeners were able to detect LFS (32 Hz) and IS (8 Hz) tone bursts in a sound booth as well as in the MRI scanner and could evaluate them in terms of perceived loudness and unpleasantness, with similar results in both environments” but unpleasantness was not assessed in the MRI environment.

**Response 2.1: It is in fact not known to which degree exactly the unpleasantness ratings in the sound booth corresponded to the implicit (sensory) unpleasantness of similar sounds in the MRI scanner. To our knowledge, studies that have systematically investigated the influence of additional background noise on unpleasantness ratings of acoustic signals, especially with respect to LFS and IS, are lacking. Given the close relationship between loudness judgments in both environments, and the use of a sparse imaging paradigm in general, it is in our view reasonable to assume that unpleasantness was comparable in both environments. However, we concede that providing data in support for this assumption would be preferred. Due to time restrictions, it was not possible to conduct both psychoacoustic tasks while participants were in the MRI scanner within one experimental session. We chose to employ the loudness scaling task rather than the unpleasantness rating task. In hindsight, we acknowledge that this decision might have biased the results in favor of the loudness estimates. Lastly, we agree that the cited statement in the Discussion is misleading.**

**To avoid misconceptions and to raise awareness of the higher uncertainty of unpleasantness estimates, we applied the following changes:**

1. **The first issue of the results summary in the Discussion (lines 453-459) has been revised: First, we removed the terms “and unpleasantness” from the statement cited by the reviewer. Second, the sentence starting in line 457 has been split into two sentences, with a few slight changes (changes are underscored):**

***“Unpleasantness ratings in the booth increased almost linearly as a function of loudness. These functions were similar for both tones […]”***

2. **The last paragraph of the discussion of the behavioral results (lines 517-523) has received a small change and an additional sentence: In line 519, it now reads:**

***“Our data indicate that loudness perception is comparable in both environments (see S1 Figure), […].”***

**This sentence has been added to the end of the paragraph:**

***“Whether the same is true with regard to the perceived unpleasantness of the LFS and IS stimuli could not be confirmed in the present study. Unlike loudness judgments, individual unpleasantness ratings were only assessed in the sound booth.”***

3. **Another sentence has been added at the end of the second paragraph in the discussion of the results concerning activation in relation to sound level, loudness and unpleasantness (line 595):**

***“Notably, as mentioned above, unpleasantness was calculated from data obtained in the sound booth, whereas loudness was also assessed in the MRI environment. Hence, unpleasantness estimates were subject to a comparatively higher level of uncertainty.”***

[2] Concerning the unpleasantness, one recent fMRI study (Staib et al., Hum Brain Mapp 2019) found a significant activation of the human primary auditory cortex for different types of sounds with a learned emotional aversiveness. Moreover, in the rodent secondary auditory cortex there have been observed neurons that selectively respond to stimuli with a learned aversive valence (Grosso et al., Nat Comm 2015). Even if not overlapping with the notion of pure sensory unpleasantness, the authors may also wish to discuss these studies.

**Response 2.2: The findings of both studies (and other related work) are in fact quite interesting. Although we argue that the participants’ ratings in our study rather reflect pure sensory unpleasantness, it appears plausible that learned aversion (due to prior events) may contribute to elevated unpleasantness ratings in psychoacoustic experiments, even for synthetic stimuli that supposedly have no emotional associations attached to them. In this case, the abovementioned studies suggest that high unpleasantness ratings would be reflected by systematic changes of activation in (primary) AC. Our data do not indicate that activation in AC was more related to unpleasantness rather than perceived loudness. Nonetheless, we decided that it is worthwhile to include the findings in the manuscript.**

**We have therefore added the following sentence in the Introduction (at line 70) of the revised version:**

***“Other studies suggest that a learned aversive valence for sounds (e.g., through fear conditioning), which might be the cause of a higher unpleasantness in some cases, is reflected by altered AC activity [e.g., 21; 22].”***

**And in the Discussion (at line XY):**

***“It is of course theoretically possible for a listener to develop a learned aversion for previously neutral sounds (as a result of past events), which might ultimately lead to elevated unpleasantness when exposed to these sounds. Findings from research in mice [21] and human listeners [22] suggest that such a learned aversive valence for acoustic signals may then be reflected by neural responses in the AC. Given that activation in AC was not better explained by unpleasantness relative to the other two variables in the present study, though, our data do not support the idea that learned aversion played an important role with respect to listeners’ unpleasantness ratings.”***

[3] The authors may also wish to include in the Discussion the perspective reported in the work of Monson, Han and Purves (PLoS ONE 2013).

**Response 2.3: The perspective reported in the work of Monson and colleagues is interesting as well. However, we decided to not discuss this work in manuscript.**

- **The article presents similarities between psychoacoustic functions and prevalence of frequencies/levels in speech (more precisely, in a speech database). The two obvious interpretations to be drawn from this are: a) Our perception is shaped by exposure to sound, or b) our speech has developed in accordance with our perception of sounds (i.e., to maximize intelligibility). Both make sense to some degree with regards to speech.**
- **What is to be concluded from this with respect to loudness and unpleasantness of LFS and IS? Here, it rather only makes sense in one direction of causality: Perception may change with daily exposure to LFS/IS. There are, in fact, researchers that have provided anecdotal evidence in favor of this idea (e.g., residents in proximity of wind turbines report higher levels of annoyance/unpleasantness to LFS/IS).**
- **Still, we argue that this information takes the reader too far away from the main topic of the present study, that is, the exploration of neural correlates of perception. Our participants might have differed in terms of perception due to several factors, including prior exposure to LFS. In fact, the influence of these and other factors are explored in another article that investigates data from the questionnaire that was filled out by our participants and many more at other locations. However, in our view this information does not really help elucidate why these differences in perception were related (or not) to fMRI activation.**

[4] One typo: “resented” instead of “presented” (line 187).

**Response 2.4: This typo has been fixed.**
